# Effects of Baseline Blood Zinc Levels on the Humoral Immune Response After COVID-19 mRNA Vaccination: A Prospective Study in a Japanese Population

**DOI:** 10.3390/vaccines12121359

**Published:** 2024-11-30

**Authors:** Mohammad Said Ashenagar, Megumi Hara, Gouki Yamada, Mikiko Tokiya, Akiko Matsumoto

**Affiliations:** 1 Department of Social and Environmental Medicine, Faculty of Medicine, Saga University, 5-1-1 Nabeshima, Saga 849-8501, Japan; sx4932@cc.saga-u.ac.jp; 2 Department of Preventive Medicine, Faculty of Medicine, Saga University, 5-1-1 Nabeshima, Saga 849-8501, Japan; harameg@cc.saga-u.ac.jp; 3 The United Graduate School of Agricultural Sciences, Kagoshima University, 1-21-24 Korimoto, Kagoshima 890-0065, Japan; k6658152@kadai.jp

**Keywords:** humoral immune response, immunogenicity, SARS-CoV-2, vaccination, zinc

## Abstract

Background/Objectives: Although the protective effects of zinc against COVID-19 are documented, its impact on COVID-19 vaccine immunogenicity remains unknown. Methods: We conducted a prospective study involving a cohort of 79 Japanese individuals (aged 21–56 years; comprising three subcohorts) and measured their serum zinc levels pre-vaccination and anti-SARS-CoV-2 IgM/IgG levels pre- and post-vaccination over 4 months. Results: Serum zinc concentrations ranged between 74–140 and 64–113 μg/dL in male and female individuals, respectively, with one male and 11 female participants exhibiting subclinical zinc deficiency (60–80 μg/dL). Mixed models for antibody titers, accounting for the subcohorts, repeat measurements, and covariates (e.g., vaccine type, sex, age, height, steroid use, medical history, smoking and drinking habits, perceived stress, and sleep disturbances) showed positive effects of zinc on IgM (*p* = 0.012) and IgG (*p* = 0.013) in 45 female individuals with 255 observations. However, a similar association was not found in the 34 male participants with 162 observations. This discrepancy may be attributed to one participant being included in the subcohort with frequent repeat measurements (10 repeats in 4 months). COVID-19 mRNA vaccine immunogenicity was enhanced in the participants with high baseline blood zinc levels within the reference range. Conclusions: Our findings underscore the relevance of maintaining adequate zinc levels before vaccination, which can be achieved through a balanced diet and healthy lifestyle choices.

## 1. Introduction

COVID-19 is a global health threat, highlighting the crucial role of the immune system in combating the SARS-CoV-2 infection. Recognized risk factors for severe infection include age, sex, and insufficient levels of micronutrients, including zinc [1,2,3,4]. Zinc, an essential trace element, plays a vital role in supporting both innate and adaptive immunity [5,6,7,8]. For instance, zinc is involved in the maturation of dendritic cells, the activation of mast cells, and T cell maturation. The disruption of zinc homeostasis can profoundly affect the adaptive immune response [9], potentially impairing antibody production [10], which crucially affects the humoral immune response, also known as the antibody-mediated immune response [11]. Given its crucial role in immune function, it is essential to investigate the association between baseline zinc levels and vaccine immunogenicity.

According to an observation of elderly individuals in nursing homes in the United States, normal baseline concentrations of blood zinc have been linked to lower overall mortality rates compared with low levels of zinc [12]. Several studies have reported low serum zinc levels in patients with COVID-19, which are associated with severe disease, death risk, and poor clinical outcomes [13,14,15]. Moreover, other studies have suggested that zinc supplementation could contribute to an additional defense against the illness [16,17,18], potentially by lowering the viral load and enhancing the immunity of patients with COVID-19. However, the association between the baseline levels of serum zinc and post-vaccination antibody titers has been scarcely studied. In an observational study of healthcare workers in Germany with a moderate level of baseline zinc who received two doses of an anti-SARS-CoV-2 vaccine (BNT162B2), the total serum zinc levels and supplementation showed no significant associations [19]. We believe that the results are inconclusive due to limitations, such as the small number of observation points, participants’ ethnicity, and confounding factors, such as psychological stress, sleep deprivation, and physical exercise, which could affect the zinc levels [20] and immune system function. Therefore, we performed a prospective study in a Japanese general population, measuring the serum zinc levels prior to vaccination and tracking anti-SARS-CoV-2 IgM and IgG levels before and for 4 months following vaccination. Our aim was to examine how baseline blood zinc levels affect the humoral immune response following COVID-19 mRNA vaccination.

## 2. Materials and Methods

This study was approved by the Ethics Committee for Clinical Research (approval number: R2–44; date of approval: 25 March 2021, R3–9; date of approval: 28 April 2021, R3–39; date of approval: 24 November 2021) and the Human Genome/Gene Research Ethics Committee of Saga University (approval number: R2–24; date of approval: 25 March 2021, R3–4; date of approval: 28 April 2021). Written informed consent was obtained from all participants prior to any study-related procedures.

### 2.1. Study Design and Participants

**Figure 1:**
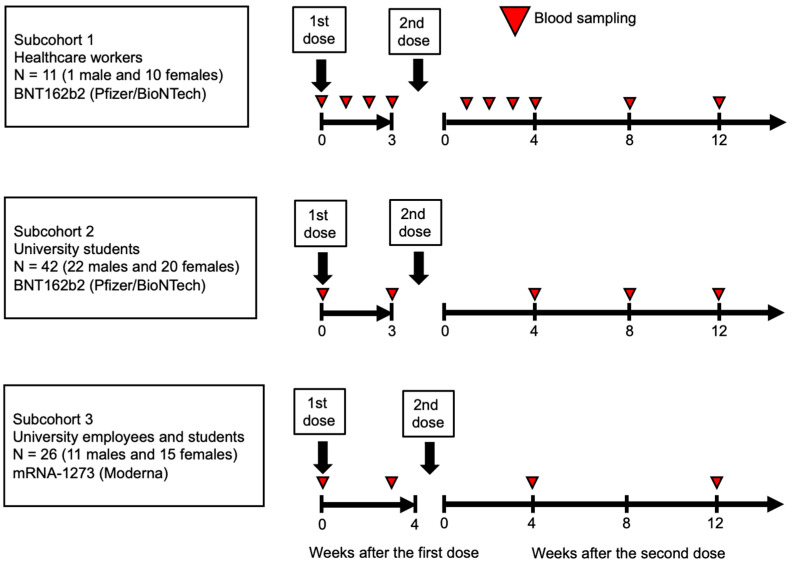
Study design. All participants, including healthcare workers, university students, and employees, received two doses of the Pfizer and Moderna (BioNTech) COVID-19 vaccines. Blood samples were collected at Week 0 and at various intervals up to Week 12 after the second vaccination from healthcare workers (Subcohort 1), university students (Subcohort 2), and university employees and students (Subcohort 3).

A total of 79 participants, comprising 11 healthcare workers, 68 students, and employees from hospitals and a university in Saga Prefecture, were enrolled in this study. Participants received either two doses of BNT162b2 (Pfizer Inc., New York, NY, USA/BioNTech SE, Mainz, Germany) at a dose of 30 µg (N = 53) or mRNA-1273 (Moderna Inc., Cambridge, MA, USA/Takeda Pharmaceutical Co., Ltd., Tokyo, Japan) at a dose of 100 µg (N = 26) (Figure 1). The total number of observations was 255 for 45 female and 162 for 34 male individuals. The first and second doses of BNT162b2 were administered between April and May 2021, with a 21-day interval between doses. The first and second doses of Moderna’s mRNA-1273 were administered in August 2021, with a 28-day interval between doses. None of the participants had a history of COVID-19 prior to vaccination. Blood samples were collected from all participants before the first vaccination (Week 0) and at various intervals up to Week 12 after the second vaccination. This included healthcare workers (Subcohort 1), university students (Subcohort 2), and university employees and students (Subcohort 3).

### 2.2. Laboratory Testing

Serum samples were processed on the day of blood collection and subsequently stored at −80 °C for later analysis. To quantify the levels of three types of anti-SARS-CoV-2 antibodies—anti-spike protein (S1) IgG, anti-S1 IgM, and anti-nucleocapsid protein (N) IgG—a high-sensitivity chemiluminescent enzyme immunoassay (CLEIA) platform (Sysmex Co., Kobe, Japan) was utilized [21]. The antibody concentrations were expressed as binding antibody units per milliliter (BAU/mL) for anti-S1 IgG and Sysmex units per milliliter (SU/mL) for both anti-S1 IgM and anti-N IgG. The BAU measurements were standardized based on the WHO International Standards.

### 2.3. Self-Administered Questionnaire

Data were collected using a self-administered questionnaire, which included details of participants’ sex, age, height, weight, smoking habits, alcohol consumption, exercise routines, perceived stress, sleep disturbances, and medical history. Smoking behaviors were reported as unchanged over the past year. Alcohol intake was estimated based on the amount consumed in the preceding six months, adjusted for a 60 kg body weight, and categorized into four levels: less than 1 g/day, 1–20 g/day, and 20 g/day or more. Exercise routines were evaluated through the question, “Do you typically engage in physical activity?” with responses categorized as: no regular activity, less than once per week, 1–3 times per week, or more than three times per week. Stress levels were rated on a five-point scale, with options ranging from none (0) to yes, frequently (4). Sleep disturbance was classified as “yes” for positive indications and “no” for no indications. Participants receiving steroids at the time of the study were classified as “yes” for steroid use, while those who had not used steroids within the preceding 3 years were considered as “no.” Dyslipidemia was similarly categorized: participants with concurrent dyslipidemia were marked as “yes”, while those without a history of dyslipidemia in the previous three years were marked as “no”.

### 2.4. Statistical Analyses

**Figure 2:**
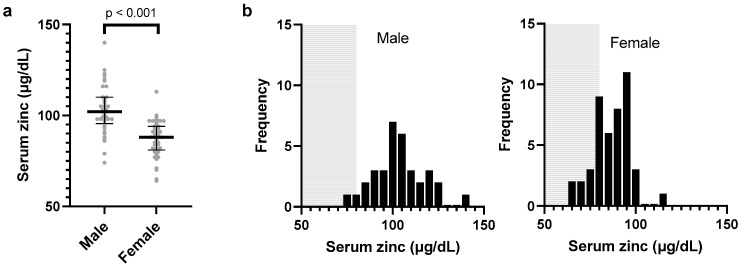
Zinc levels according to sex and distribution. The total blood zinc levels are displayed as dots for each individual, categorized by sex (male and female). The bars represent the median zinc levels, and the error bars indicate the interquartile range. The *p*-value was used for Spearman’s rank correlation between sex and zinc levels (**a**). The histograms (**b**) represent the distribution of zinc levels separately for male and female individuals. The gray-shaded area corresponds to zinc deficiency (<80 μg/dL) according to the general standards in Japanese hospitals, allowing for the comparison of the zinc deficiency prevalence between sexes.

Statistical analyses were performed using SAS 9.4 TS Level 1M5 for Windows (SAS Institute, Cary, NC, USA) with an alpha value of 0.05. The primary outcomes, anti-S1-IgG (BAU/mL) and anti-S1-IgM (SU/mL) levels, along with the serum zinc level (μg/dL) as the primary explanatory variable, were log-transformed to achieve an approximate normal distribution. Male and female individuals were examined separately for the following reasons: (1) the estimation was possible for the whole observation period for 45 female individuals (255 observation points), while data for 34 male participants (162 observation points) were non-estimable at 1–3 weeks after the second dose; and (2) there was a significant difference in the zinc levels according to sex (Figure 2).

#### 2.4.1. Covariates

The analysis incorporated several covariates alongside sex, age, vaccine type, and weeks post-vaccination. These included height, smoking habits, alcohol consumption, exercise frequency, perceived stress, sleep issues, steroid use, and the presence of dyslipidemia. Previous research has identified these factors as being linked to variations in vaccine effectiveness, immune response, and zinc levels [22,23,24,25,26,27,28,29].

#### 2.4.2. Mixed Model

Mixed model analysis was conducted to estimate log-transformed antibody titers, accounting for subcohorts, repeat measurements, and covariates in three models. Model 1 included the effects of the post-vaccination week, vaccine type, age, and zinc levels. Model 2 included lifestyle habits and current medical history factors, such as body height, steroid use, dyslipidemia, allergic disease, exercise habits, cigarette smoking, ethanol intake, perceived stress, and sleep disturbance.

#### 2.4.3. Least Squares Geometric Means

The non-linear dose–response relationships between zinc, anti-S1-IgG, and anti-S1-IgM are depicted as least-squares geometric means and standard errors for the categorized zinc levels. Anti-S1 IgG and IgM levels corresponding to T1–T3 groups are visualized using Model 1, with modification for zinc. The term “log-transformed zinc” was replaced by “T1–T3 × time course” to reflect the interaction between zinc levels (T1–T3 groups) and the time course.

#### 2.4.4. Sensitivity Analysis

Among the 45 female participants, one with dyslipidemia was excluded from the sensitivity analysis, resulting in 44 participants with a total of 245 data points. When conducting additional analyses, we considered body weight instead of height.

## 3. Results

### 3.1. Baseline Characteristics

During the observation period, all participants had anti-N IgG levels below 1.2 SU/m, indicating no previous exposure to the virus or vaccination. Table 1 shows the baseline characteristics of the 79 participants. The study group comprised 45 female (57%) and 34 male participants (43%) with an age range of 21–56 years. The baseline characteristics of the participants are presented in Table 1, which categorizes them into three quantiles (T) based on baseline serum zinc levels by sex. The distribution of exercise habits and perceived stress did not differ among the tertiles (female, *p* > 0.6; male, *p* > 0.8, Fisher’s exact test). Two male participants in the T3 group reported steroid use, whereas one female participant in the T1 group reported dyslipidemia.

### 3.2. Effect of Baseline Zinc on Anti-S1 IgM Post-Vaccination

**Figure 3:**
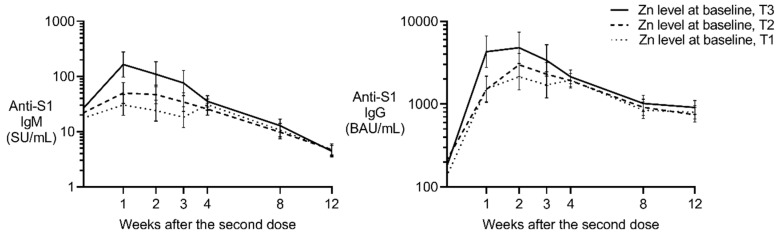
Estimated anti-S1 IgM and IgG levels after the second dose in female participants. Least-squares geometric means and standard errors were calculated using mixed models that account for vaccine type, age, and the interaction between zinc levels (T1, T2, and T3 as class variables) and the number of weeks post-vaccination (as a class variable). The models include random effects based on repeated measures and the three cohorts. Binding antibody units (BAU) were calibrated against the WHO international standard, and the x-axis is displayed on a log-2 scale for improved visualization. SU, Sysmex unit.

Although no association between zinc and anti-S1 IgM was found in male participants, a positive effect was observed in female participants. The partial regression coefficient (β) for log-transformed zinc on log-transformed anti-S1 IgM (SU/mL) was 2.2 (*p* < 0.001) and 1.5 (*p* = 0.012) in Models 1 and 2, respectively (Table 2). As shown in Figure 3 (left panel), anti-S1 IgM levels exhibited the most significant differences at 1–3 weeks after the second dose. Participants in the highest zinc tertile (T3; 93–113 μg/dL) displayed the highest anti-S1 IgM levels compared to those in the lower tertiles (T1 and T2; 64–92 μg/dL). The area under the curve (AUC) of T3 was 2.4 times greater than that of T1.

### 3.3. Effect of Baseline Zinc on Anti-S1 IgG Post-Vaccination

Similarly to IgM, the positive fixed effect was estimated in females with a β of 1.7 (*p* < 0.001) in Model 1 (Table 3). The positive effect of zinc in female individuals remained after the inclusion of lifestyle habits and current medical history (β = 1.1, *p* = 0.013 in Model 2). Similarly to the IgG response, anti-S1 IgG levels differed most significantly 1–3 weeks after the second dose (Figure 3). The highest zinc tertile displayed the highest anti-S1 IgG levels compared to those in the lower tertiles, suggesting a beneficial effect of higher pre-vaccination zinc levels on the post-vaccination humoral immune response. The AUC of T3 was 1.6 times greater than that of T1.

### 3.4. Sensitivity Analysis

Sensitivity analysis, excluding one female participant with dyslipidemia (44 female individuals; 245 observations), resulted in a similar estimation (AIC = 659, β = 1.5, *p* = 0.013 in Model 2 for IgM, and AIC = 512, β = 1.1, *p* = 0.013 in Model 2 for IgG). A slightly modified Model 2, including weight instead of height, produced similar estimates (AIC = 681, β = 1.4, and *p* = 0.018 in Model 2 for IgM, and AIC = 536, β = 1.1, and *p* = 0.013 in Model 2 for IgG).

## 4. Discussion

Our prospective study investigated the impact of baseline blood zinc levels on the humoral immune response and production of anti-S1 IgG and IgM antibodies following COVID-19 mRNA vaccination in a Japanese cohort. Using mixed models considering repeated measures and covariates, we found higher post-vaccine IgG/IgM production in female participants with higher baseline zinc levels within the normal range, suggesting a positive effect of adequate zinc levels on optimal immune system activity. Such an association was not found in male participants, probably because only one participant was included in the subcohort with frequent repeated measurements (10 repeats in 4 months).

Zinc may increase antibody titers via various pathways [30]. For example, zinc may increase the downstream signal interleukin-2 by maintaining the three-dimensional structure of NFκB, thereby increasing the production of IgG [31] and IgM [32]. Additionally, zinc is crucial for regulating T follicular helper (Tfh) cell production and plays a vital role in humoral immunity [9]. The zinc-dependent transcription factor, B-cell lymphoma 6 (BCL6), is particularly important, as it requires zinc for structural integrity to support B-cell maturation within germinal centers. BCL6 regulates B cell and Tfh cell interactions, facilitates antibody class switching, and promotes humoral immunity by upregulating key downstream proteins [33].

Our finding of a significant correlation between baseline zinc levels and antibody response to mRNA vaccination is inconsistent with that of a previous study conducted on a German population [19]. One of the advantages of our design over that of this study is the inclusion of multiple covariates; however, multivariate adjustment did not affect this association. The strong impact observed 1–3 weeks after the second dose in our study suggests that the frequent observation points played a crucial role in detecting significant associations. In contrast, the previous study lacked observations within the first month post-vaccination [19], whereas we conducted four observations within this period.

### Limitations

This study had a small sample size with a convenience sample, particularly among male participants, which limits the generalizability of the results. Additionally, the small sample size limits the power to detect significance detection for each observation time point over a wide range, which limits the interpretation of the results. For example, based on our observation, a sample size of 400 or more would be required to detect a significant association in the female T1–3 groups at 2 months after the second dose (with a power of 0.8 and an alpha error of 0.05). Given the small sample size, these findings need to be validated in larger cohorts. However, with the widespread spread of COVID-19, conducting similar prospective studies in human populations may no longer be feasible. Future efforts should focus on leveraging remaining samples from existing cohorts and conducting confirmatory animal studies to further investigate the relationship between baseline zinc levels and the immune response to COVID-19 vaccination. Additionally, as blood zinc levels would be transiently elevated when humoral immunity is activated [19], there is a possibility of misclassifying baseline zinc levels due to the infectious status of the participants. However, as the participants in this study were in a healthy state suitable for receiving the COVID-19 vaccine, we consider this possibility to be less likely. Finally, the uncertainty of the clinical impact should be addressed. According to our observations, the AUC for IgM and IgG in the T3 group were 2.4-fold and 1.6-fold higher than those in the T1 group, suggesting that the effect on IgM may be greater, but further confirmation is needed.

## 5. Conclusions

Our prospective study reported a positive association between baseline blood zinc levels and humoral immune response to COVID-19 vaccination in a Japanese population. This finding suggests the importance of assessing blood zinc prior to vaccination, as managing zinc concentrations through appropriate dietary and lifestyle modifications may enhance vaccine efficacy.

## Figures and Tables

**Table 1:** Baseline characteristics of the participants.

	Male			Female		
Serum zinc categories	T1	T2	T3	T1	T2	T3
Median (μg/dL)	90.5	103	119	78.5	88.5	97
Interquartile range (μg/dL)	74–98	99–107	108–140	64–82	83–92	93–113
Number	12	11	11	16	14	15
Age, years						
Median	23	22	23	37.7	22.2	22
Interquartile range	20.7–63.0	20.5–47.2	20.9–46.8	22.0–59.0	21.3–54.0	20.7–59.6
Occupation						
Healthcare worker	1	0	0	3	5	2
Student	7	7	8	6	6	8
University worker	4	4	3	7	3	5
Smoking, yes	0	1	0	0	0	0
Steroid use, yes	0	0	2	0	0	0
Dyslipidemia	0	0	0	1	0	0
Perceived stress *						
0/1/2/3/4	3/1/3/5/0	5/1/1/3/1	5/1/3/2/0	2/2/5/5/2	4/1/4/4/1	6/1/2/5/1
rs671 variant allele **						
0/1/2	8/3/1	3/5/3	4/5/2	10/6/0	7/6/1	10/4/1
Ethanol, g/day/weight60kg						
<1/≥1, <20/≥20	3/8/1	6/4/1	6/5/0	12/4/0	7/5/2	11/4/0
Exercise habit ***						
0/1/2/3	2/4/4/2	2/5/2/2	3/0/5/3	7/1/5/3	6/0/5/3	10/1/3/1
Allergic disease, yes	2	0	2	4	1	1
Sleep disturbance	0	2	0	0	1	1

* No (0) to yes (4). ** ALDH2*1/*1 (0), ALDH2*1/*2 (1), ALDH2*2/*2 (2). *** No habit (0), less than once a week (1), 1 to 3 days/week (2), and more than 3 days/week (3).

**Table 2:** Estimation of fixed effects on log-transformed anti-S1 IgM (SU/mL).

	Males				Females			
	Model 1		Model 2		Model 1		Model 2	
	AIC = 427.1		AIC = 419.6		AIC = 682		AIC = 680	
	162 observations		162 observations		255 observations		255 observations	
	34 participants		34 participants		45 participants		45 participants	
Fixed effect	β	*p*	β	*p*	Β	*p*	β	*p*
BNT162b2 (reference)								
mRNA-1273	−0.05036	0.7928	−0.2172	0.3295	0.251	0.1028	0.239	0.2248
Age (per year of age)	−0.01927	0.0327	−0.00379	0.6982	−0.00958	0.0573	−0.005	0.4755
Body height (per cm)			−0.01749	0.2796			−0.012	0.3177
Steroid use, yes			−1.1015	0.0055			—	—
Dyslipidemia, yes			—	—			−1.3556	0.0002
Allergic disease, yes			0.1747	0.3552			0.005	0.9729
Exercise habit (per category)			0.01473	0.8767			0.028	0.6047
Cigarette smoke, yes			−0.07372	0.9281			—	—
Ethanol intake (per category)			−0.5076	0.0008			0.2721	0.0139
Perceived stress (per category)			−0.02271	0.7359			0.0276	0.6176
Sleep disturbance, yes			−0.3611	0.5297			−0.7167	0.0124
Log zinc (μg/dL)	−0.7695	0.1856	−0.4191	0.4909	2.2135	<0.0001	1.5435	0.0122

The impact of baseline characteristics was analyzed using mixed models that account for repeated measurements and the random effect of subpopulations. All models incorporated the fixed effects of the post-vaccination week as a categorical variable, along with the variables shown in the table. The findings are presented in Sysmex units per mL (SU/mL). β represents the partial correlation coefficient.

**Table 3:** Estimation of fixed effects on log-transformed anti-S1 IgG (BAU/mL).

	Males				Females			
	Model 1		Model 2		Model 1		Model 2	
	AIC = 258		AIC = 263		AIC = 529		AIC = 535	
	162 observations		162 observations		255 observations		255 observations	
	34 participants		34 participants		45 participants		45 participants	
Fixed effect	β	*p*	β	*p*	β	*p*	β	*p*
BNT162b2(reference)								
mRNA-1273	0.4599	0.0062	0.3233	0.0094	0.8347	<0.0001	0.7555	<0.0001
Age (per years of age)	−0.012	0.017	−0.005	0.3195	−0.005	0.1384	−0.0038	0.4312
Body height (per cm)			−0.0165	0.0659			−0.002	0.8262
Steroid use, yes			−0.4357	0.0455			—	—
Dyslipidemia, yes			—	—			−0.6725	0.0107
Allergic disease, yes			0.1581	0.131			−0.2301	0.0196
Exercise habit (per category)			0.0388	0.46			−0.0102	0.7973
Cigarette smoke, yes			−1.2462	0.0064			—	—
Ethanol intake (per category)			−0.1627	0.0487			−0.0068	0.9326
Perceived stress (per category)			0.07533	0.0444			0.044	0.2769
Sleep disturbance, yes			−0.7196	0.0246			−0.388	0.064
Log zinc (μg/dL)	−0.3268	0.3113	−0.056	0.8676	1.609	<0.0001	1.124	0.0131

The influence of baseline characteristics was assessed using mixed models, which accounted for repeated measurements and the random effect of the subpopulation. Each model included post-vaccination week as a categorical fixed effect, along with the variables presented in the table. BAU (binding antibody units) were calibrated according to the WHO International Standard. β represents the partial correlation coefficient.

## Data Availability

Data presented in this study are available upon request from the corresponding author (A.M.). The data are not publicly available due to privacy concerns.
